# Reevaluation of motoneuron morphology: diversity and regularity among motoneurons innervating different arm muscles along a proximal–distal axis

**DOI:** 10.1038/s41598-020-69662-z

**Published:** 2020-08-04

**Authors:** Satoshi Fukuda, Hitoshi Maeda, Masaki Sakurai

**Affiliations:** Department of Physiology, Teikyo University School of Medicine, Kaga 2-11-1, Itabashi-ku, Tokyo, 173-8605 Japan

**Keywords:** Cellular neuroscience, Motor control, Neural circuits, Neuronal physiology, Neuroscience

## Abstract

Dendritic fields of spinal motoneurons (MNs) are popularly believed to be stellate; however, variation in dendritic arborization, especially concerning the innervated muscle groups, has not been systematically studied. We addressed this problem by injecting Neurobiotin through patch-pipettes into single MNs (rat cervical cord slices) that had been retrogradely labelled from innervated muscle groups situated along a proximal (here named “scapular” including serratus anterior) to distal (forearm) axis. MNs had fairly straight dendrites with small numbers of mainly proximal branches that exhibited acute branch angles, leaving large areas around the cells with no dendrites. MNs in the same group of pools showed similar morphologies, but there were clear differences among groups. Forearm MNs (n = 35) showed hemidirectionally-extended multipolar dendrites, whereas scapular MNs (n = 15) had bipolar dendrites, and pectoralis MNs (n = 20) had an intermediate morphology. MNs thus showed a spectrum of morphologic characteristics along the axis. This may be because more distally-located forearm muscles are involved in finer movements and need a wider variety of inputs for neural control than proximally-located muscles. We also devised a quantitative method for evaluating the degree to which a cell’s dendritic field displays a symmetric spherical shape; only 20% of MNs tested reached this criterion.

## Introduction

Spinal motoneurons (MNs) are one of the most studied classes of neurons in the mammalian central nervous system. Numerous studies have been published on their morphology^1–8^, which is generally regarded as stellate or star-shaped with dendrites extending radially in all directions from the cell body (“stellate or cylindrical”^3^, “triangular”^9^, “star-shaped”^9,10^. The somata of MNs innervating each muscle are arranged in a cluster within the spinal cord (MN pool)^3,11^. Several studies have shown that the morphology of MNs varies depending on the cell’s location within the spinal ventral horn^2,8,12^, which may reflect morphological differences among pools. There have also been studies in which MN somata and their proximal-most dendrites were retrogradely labeled and examined^4,5,9,12^. However, systematic investigation of the total morphology of individual MNs within defined groups of MN pools has yet to be done. In the present study, we identified MN somata in cervical spinal cord slices within their pool groups after retrogradely labeling them with tracer from the muscles that they innervate. We injected tracer into three groups of muscles controlling forearm movements along a proximal–distal axis; that is, from proximally-located muscles involved in coarse movement to distally-situated muscles regulating fine movement. The injected muscles were the “scapular” muscles including serratus anterior muscle, pectoralis muscles, and forearm muscles including extensor carpi radialis, extensor digitotum communis, palmaris longus, flexor digitorum profundus, and flexor carpi radialis (see “Methods” section for details). Subsequently, Neurobiotin was directly injected into the target MNs from patch pipettes^13^, which enabled us to study the entire morphology of each MN. We also endeavored to quantitatively analyze the arborization pattern of MN dendrites and to determine how similar each MN’s morphology was to being stellate.

## Results

### Morphological characteristics of cervical MNs

**Figure 1:**
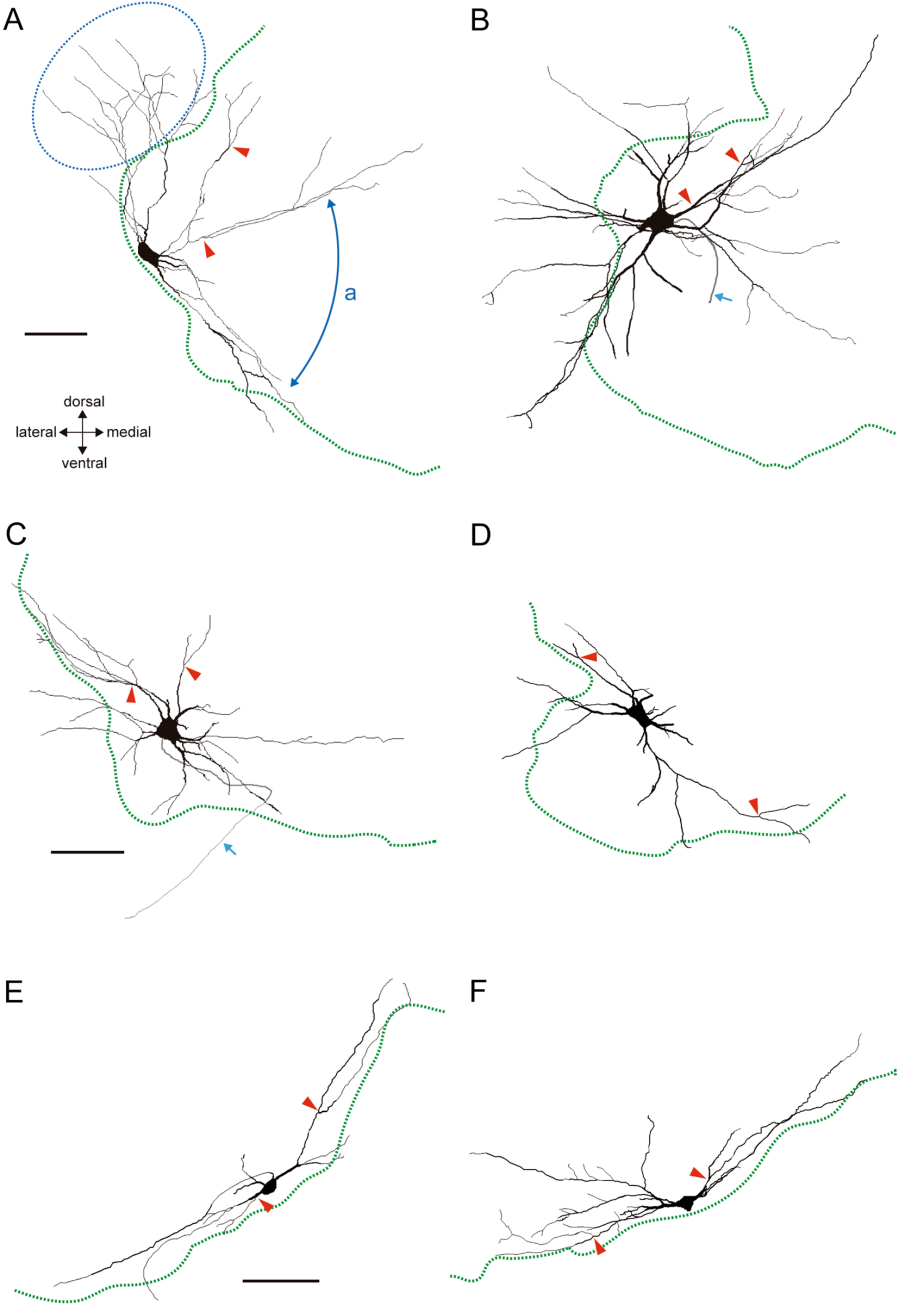
Neurobiotin-injected MNs traced with Neurolucida software version 11.01.2: forearm (**A**, **B**), pectoralis (**C**, **D**) and scapular (**E**, **F**). Red arrowheads point to Y-shaped bifurcations with acute angles; pale blue arrows point to axons. Dendrites invading the white matter are indicated by a blue dotted circle (**A**). The pale blue double headed arrow indicates a gap (*a*). The dotted green lines indicate the boundary between white and gray matter. Scale bar = 100 μm.

Representative examples of three groups of MNs innervating the forearm, pectoralis and scapular muscles labelled with intracellularly-injected Neurobiotin are shown in Fig. 1. In all three groups, dendritic branching usually entailed symmetrical Y-shaped bifurcations producing sister dendrites branching at acute angles (red arrowheads in Fig. 1A–F), rather than branching where a smaller (daughter) dendrite sprouted from a larger stem (mother) dendrite. Once dendritic branching occurred at a proximal site (< 150 μm from the soma), the frequency of branching decreased, which reduced the solid angle of the conical radiation of the dendritic arborization (Fig. 1A). The somata of the MNs tended to be located within a narrow zone along the white–gray matter boundary (dotted green lines in Fig. 1A–F) in the ventral horn. Dendrites generally did not invade the white matter, which left a large area that lacked dendrites located in the direction of the white matter (Fig. 1A, E, F). For these reasons, we often found gaps devoid of dendrites around MN somata (Fig. 1A, an arc with double arrowheads, D–F).

**Figure 2:**
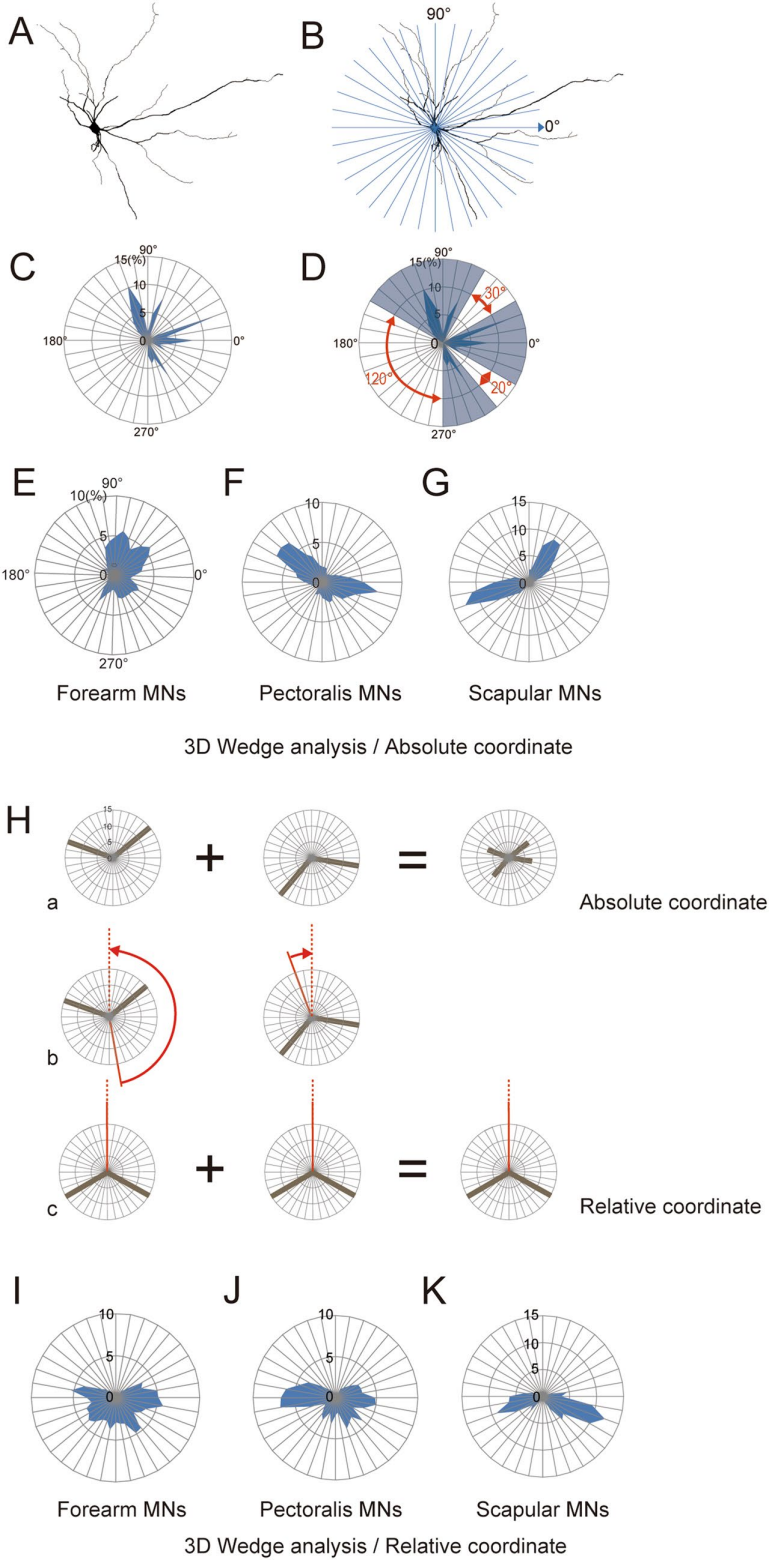
Wedge analysis of MN morphology. (**A**) A forearm MN traced using Neurolucida version 11.01.2. (**B**) The soma in A was centered, and the dendritic field was divided into 36 parts for wedge analysis. On absolute coordinates, angles were measured in a counterclockwise direction from the medial horizontal line (0°). (**C**) Polar diagram generated with the wedge analysis shown in (**B**). The ratio of dendritic length in each fraction was normalized to the full length of the dendrites in each MN. Their normalized length (expressed as a %) was plotted on the counterclockwise side of the two lines defining each fraction, which were connected by straight lines. (**D**) “Gaps” (between blue fan-like areas) were defined as angles of consecutive fractions that contained less than 1% of the total length of the dendrite indicated by the red arcs. (**E**–**G**) Polar diagrams of corrected data for each of the three MN pool groups presented on absolute coordinates: forearm MNs (**E**), pectoralis MN (**F**) and scapular MN (**G**). (**H**) (a) If two MNs with the same pattern of dendritic arborization, but different orientations (left side) are simply summed in this co-ordinate system, the result (right side) would produce a pattern different from their original ones. (b, c) However, when the midlines at half the maximum gaps (red line) are rotated to be 0° (red dotted line) (b), the summed figure of these two reproduces the original pattern (c, relative coordinates). (**I**–**K**) Polar diagrams of the three groups of MNs presented on relative coordinates: forearm MNs (**I**), pectoralis MNs (**J**) and scapular MNs (**K**).

Although MNs within each of the three groups had similar morphologies, there were noteworthy differences among the three groups (Figs. 1, 2, 4C). The arborization pattern of scapular MN dendrites showed a relatively simple bidirectional pattern (Fig. 1E, F). On the other hand, forearm MNs exhibited a multipolar dendritic tree (Fig. 1A, B). Pectoralis MNs showed intermediate patterns between those of the scapular and forearm MNs (Fig. 1C, D).

### MNs innervating distal (forearm) muscles

We confirmed that the somata of forearm MNs are located on the dorsolateral side of the ventral horn of the cervical cord (n = 35, Fig. 1A, B) (rat^17^, mouse^18,19^). When the somata were situated away from the white–gray matter boundary (i.e. when surrounded by a relatively large area of gray matter), the dendritic arborization exhibited the multidirectional pattern. However, when their positions were near the white–gray mater boundary (Fig. 1A), their dendritic extensions were limited by that boundary, though in a few cases dendrites extending into the white matter (Fig. 1B). Thus, forearm MN dendritic fields are largely multipolar with a hemidirectional bias produced by avoiding the white matter (Fig. 1A). Curiously, a part of the lateral funiculus interposed between the dorsal edge of the ventral horn and the ventral edge of the dorsal horn appeared not to follow this rule. There, for unknown reasons, MN dendrites freely invaded the white matter. The extension of dendrites into this region is indicated by a dotted blue line in Fig. 1A. All twelve forearm MNs that extended their dendrites to the dorsal side extended them directly into the dorsal white matter. Further examination of the inputs to these MNs may provide a clue to why the border is ignored in this case.

### MNs innervating proximal (pectoralis, scapular) muscles

The scapular MNs (n = 15, Fig. 1E, F) studied were located within a narrow area ventromedially between the ventral horn and the white matter. These MNs did not extend their dendrites toward the center of the gray matter or into the white matter. Instead, the dendrites extended in both directions within a narrow region along the white–gray matter boundary. This rendered the dendritic fields elongated in the horizontal dimension (Fig. 1E, F).

The somata of pectoralis MNs (n = 20, Fig. 1C, D) were located on the ventrolateral side of the ventral horn. Pectoralis MNs displayed an intermediate morphology. They had fewer primary dendrites and smaller dendritic fields than forearm MNs, but they had more primary dendrites and larger dendritic fields than scapular MNs. When their somata were located within gray matter (away from the white–gray matter boundary), their dendrites also often extended in the direction of the white matter. This is different from the extension preference of dendrites from scapular MNs. Pectoralis MNs also had a larger number of primary dendrites and a larger dendritic field than scapular MNs.

### Quantitative analysis of dendritic orientation

**Figure 3:**
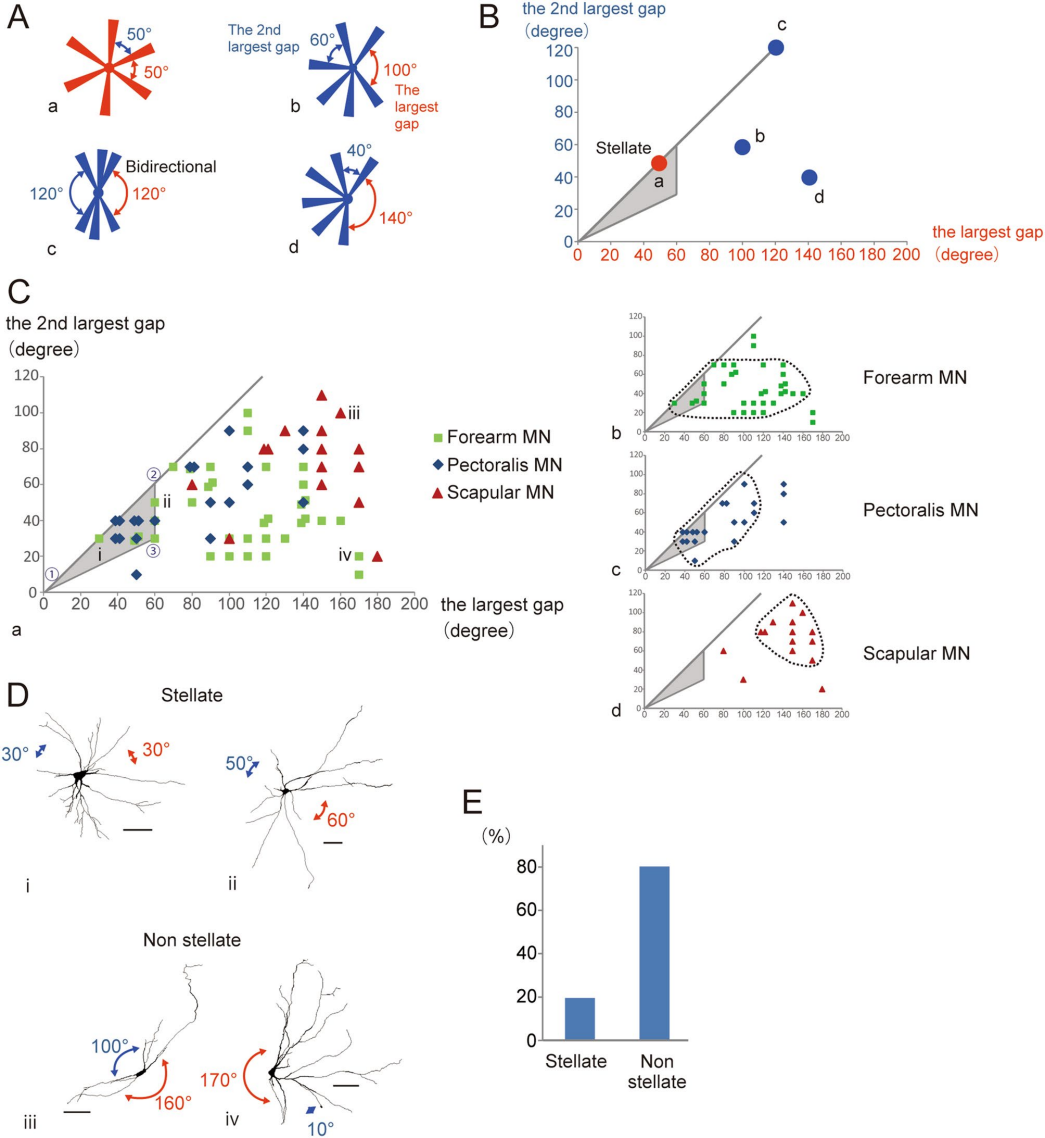
Quantitative analysis of gaps in dendritic fields: (**A**) Schematic representation of some models of dendritic morphology. a, an ideal stellate cell that radiated six dendrites equally in all directions. b, a cell with a relatively large gap. c, a bidirectional type cell. d, a hemidirectional type cell. (**B)** Illustration plotting the largest gap from the wedge analysis data on the X-axis and the second largest gap on the Y axis. The model cells in (**A**) are plotted on these coordinates (a–d). Cells within the area of the shaded triangle are considered to be “stellate.” (**C**) (a) Plot of all the MNs examined on the same type of coordinates as in (**B**). A breakdown of the three subtypes is shown to the right [forearm MNs (b), pectoralis MNs (c) and scapular MNs (d)]. (**D**) Dendritic morphology of examples of forearm MNs (i, ii, and iv) and scapular MNs (iii) in (**C**). These were traced with Neurolucida version 11.01.2. Scale bar = 100 μm. (**E**) Ratio of “stellate” and “not stellate” MNs classified using the present criteria (see text).

To study the orientation of dendrites with respect to the somata, wedge analysis was carried out. The traced data were projected onto a map of the transverse plane of the spinal cord (Fig. 2A). MNs located on the right side of the spinal cord were geometrically turned over the midline so that their mirror images could be obtained with the Neurolicida software. Thus, we analyzed the morphology of these MNs as if they were located on the left side of the cord. The medial side of the horizontal line was set to 0° with the cell body as the center, and the plane was divided into 36 spatial fractions by lines intersecting the center at an interval of 10° (Fig. 2B). The part of the dendritic length contained in each fraction was normalized to the full length of the dendrites in each MN (length of dendrites included in a fraction / total length of the dendrites × 100%). This normalized dendritic length in each fraction of the MN in Fig. 2A is presented in a polar diagram in Fig. 2C. Areas that were devoid of dendrites (i.e. containing no more than 1% of the total length of dendrites) were considered to be dendritic field gaps. This analysis enabled us to express the extent of the dendritic field gap as an arc crossing a specific angle (red arrows in Fig. 2D). An angle of consecutive blank fractions was called a “gap angle,” which was later used for the analyses in Fig. 3.

All of the data obtained in the wedge analysis of the three groups of MN pools (i.e. forearm, pectoralis and scapular) were gathered separately and displayed on separate polar diagrams (Fig. 2E–G). These three groups of MNs innervate muscles arranged along a proximal–distal axis and are thus involved in forearm movements ranging from coarse to fine. Most forearm MNs were characterized as hemidirectional and multipolar because their lateral extension was limited by the white matter (lateral funiculus), but was unaffected by the lateral funiculus on their dorsal side (Fig. 2E). Pectoralis MNs showed bidirectionality in the medial and dorsolateral directions on the ventrolateral side of the ventral horn (Fig. 2F). Scapular MNs also showed bidirectionality, but in the lateral and dorsomedial directions, along the ventromedial edge of the ventral horn (Fig. 2G). This analysis further confirms that the elongation direction of MN dendrites varies among these three MN groups, though MNs within each group share a generally similar morphology.

Although the dendritic orientations on absolute coordinates show a relationship between MNs and other spinal structures surrounding them, this system is not suitable for evaluating the pattern of dendritic arborization of individual MNs. For example, two MNs with the same dendritic pattern, but different orientations would generate another type of pattern in an analysis using absolute coordinates, as illustrated in Fig. 2H(a). To avoid this problem, we analyzed dendritic morphology by rotating each polar diagram so as to align it with reference to the largest gap (relative coordinate). Specifically, using the gap angles measured from the blank areas of the dendritic fields (Fig. 2D), the center line of the maximum gap of a cell was defined as 0°, and polar diagrams on the absolute coordinates were rotated around that center line [Fig. 2H(b)]. These relative coordinates revealed that both pectoralis and scapular MNs exhibit bidirectional characteristics (Fig. 2J, K), and that all MN groups, including forearm MNs, possess at least one wide gap in their dendritic field (Fig. 2I–K).

### Quantitative analysis of deviation from “ideal” stellate

We also attempted to quantitatively evaluate the extent to which each MN’s morphology accords with a stellate configuration. An ideal stellate cell would extend its dendrites from its soma uniformly to all directions, which means angles formed by two adjacent primary dendrites would be nearly the same [Fig. 3A(a)]. In essence, the gaps would all be about the same size in a symmetrically arranged dendritic field like that of a stellate cell. Thus, the greater the difference between the largest gap and the second largest gap, the more the cell deviates from this completely symmetrical dendritic field organization [for example, see Fig. 3A(a and b; b and d)].

We therefore plotted the largest gaps of the MNs against the second largest gaps. Figure 3A, B is provided for illustrative purposes. A cell with a single wide gap (Fig. 3A(d), hemidirectional type, such as Purkinje cells) would be located on the right side in these coordinates (d in Fig. 3B), while a cell with a roughly cylindrical dendritic field [Fig. 3A(c)] would have two large, roughly equivalent gaps, located 180° from one another, and would be plotted on upper right side (c in Fig. 3B). An ideal stellate cell (Fig. 3A(a), here the number of dendrites is arbitrarily set at six for simplicity) would be plotted on a straight line where Y = X (Fig. 3B). That cell would be located on the lower left because its gap angles would be small (a in Fig. 3B, red circle). We therefore tentatively defined the MNs located in the lower left position [a shaded area delimited by points , and (Fig. 3C)] as stellate. The three MN groups examined formed fairly characteristic clusters on this plot [Fig. 3C(b–d)]. Scapular MNs were situated in the upper right of the plot [Fig. 3C(d)] and were strongly bidirectional; none (0/15) were plotted within the area indicating them as being “stellate”. Pectoralis MNs fell mainly in a wide belt just below the Y = X line, with some (8/20) meeting the “stellate” criterion [Fig. 3C(c)]. Forearm MNs were widely distributed, including hemidirectional cells located in the lower right of the plot [Fig. 3C(b)]. Six of the 25 forearm MNs met the stellate criterion. In total, 20% of the MNs examined were classified as “stellate” (Fig. 3E).

### Comparison of other morphological parameters among MN pools

**Figure 4:**
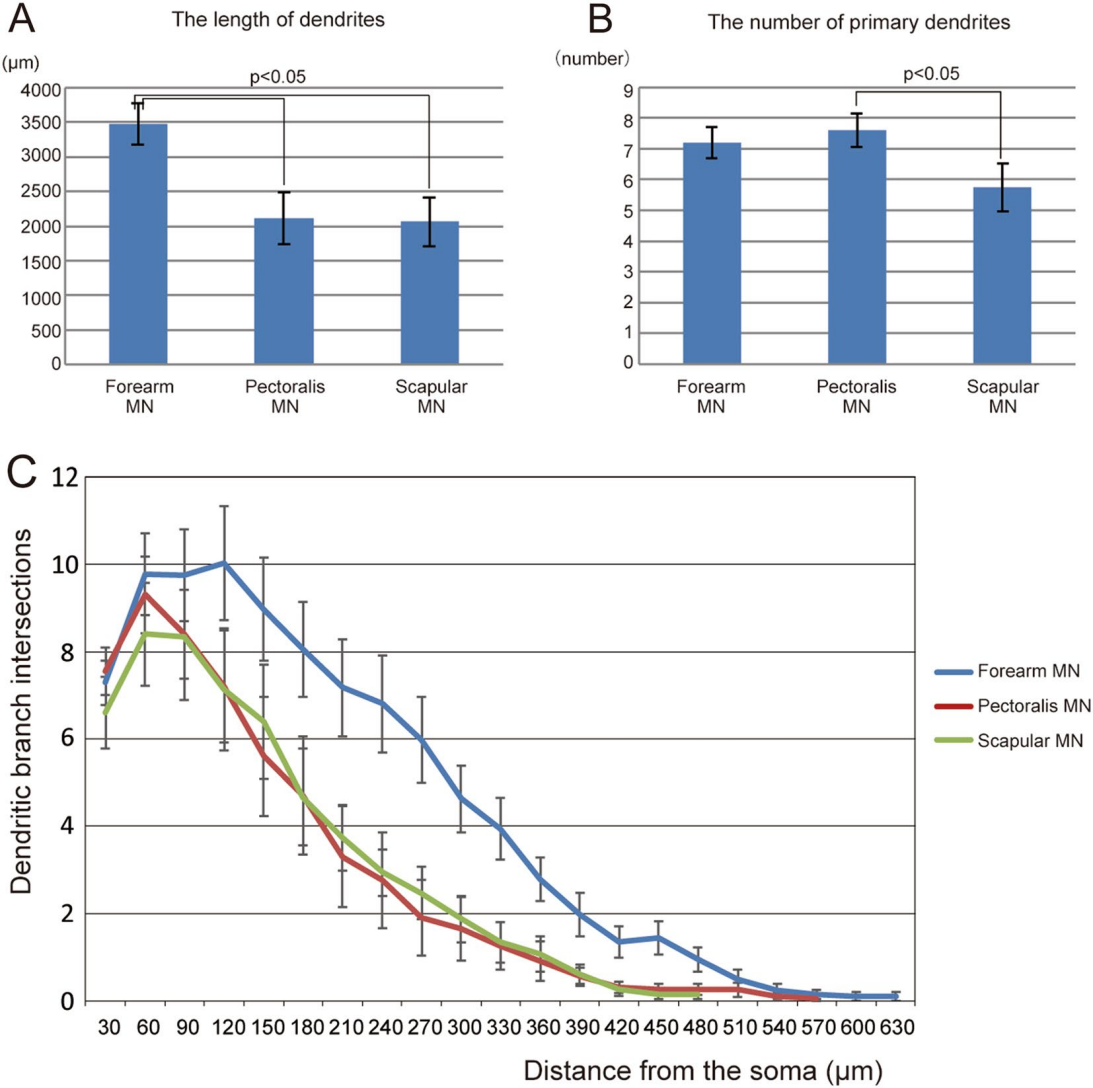
Total dendritic lengths (**A**), numbers of primary dendrites (**B**) in each MN group of pools and their Sholl analyses (**C**). In forearm MNs, the number of intersections was larger than in the other two groups (forearm vs. pectoralis between 180°–480°, forearm vs. scapular between 180°–510°, *p* < 0.05). Each bar depicts the mean ± SEM.

The dendritic length of pectoralis MNs (2,110 ± 380 μm, mean ± SEM) was similar to that of scapular MNs (2,062 ± 354 μm) (Fig. 4A), whereas the total dendritic length of forearm MNs was greater (3,478 ± 302 μm) than that of the other two groups (*p* < 0.05, Fig. 4A). Scapular MNs had somewhat fewer primary dendrites (5.7 ± 0.8) than forearm (7.2 ± 0.5) and pectoralis (7.6 ± 0.5) MNs (scapular vs. pectoralis *p* < 0.05), which were comparable (Fig. 4B). The number of primary dendrites was similar between pectoralis and forearm MNs, while the lengths of the dendrites were similar between pectoralis and scapular MNs. Here again it appears the dendritic properties of pectoralis MNs are intermediate between forearm MNs and scapular MNs.

Sholl analysis^20^ revealed that in all three groups, the number of dendritic intersections with concentric circles increased up to 120 μm from the soma and then monotonically decreased (Fig. 4C). In forearm MNs, the number of intersections was larger than in the other two groups (i.e. forearm MNs had more branches and larger dendritic fields than the other two groups).

## Discussion

The common characteristics of MNs examined in this study are as follows: (i) the dendrites had relatively few branches (Fig. 4C); (ii) the branch points were mainly located proximally (Fig. 4C); (iii) the branch angles were generally acute (red arrowheads, Fig. 1); (iv) the dendrites extended in a relatively straight fashion (Figs. 1, 4C); (v) the dendritic fields displayed at least one gap (Fig. 2I–K). MN dendritic fields in the same group of pools had similar morphologies, but there were clear differences among the groups (Fig. 2E–G). Those of forearm MNs generally showed hemidirectionality and were more symmetric (Figs. 1A, 2E), whereas, scapular MN dendritic fields were cylindrical and pectoralis MN dendritic trees displayed intermediate characteristics with respect to the symmetric nature of the field (Figs. 1C–F, 2F, G). Although the authors did not point it out in the text, in previously published Golgi-impregnation and HRP-labelled images of mammalian MNs^21–25^, these characteristics of dendritic arbors appear to be common among those species and may be the basic elementary pattern of mammalian MNs.

To observe the morphology of single MNs we injected Neurobiotin into each MN recorded in transverse slices. The images obtained using a two-photon microscope were traced three-dimensionally using Neurolucida and then converted to two-dimensional planes. Because some MNs reportedly exhibit long longitudinal dendritic extensions^26,27^, we may have missed some major dendrites extending longitudinally with respect to our transverse slices. For the following reasons, however, we expect that the large majority of our results represent the in vivo morphology of MNs with considerable reliability (see also^13^). First, in the present study, the distance from a MN’s soma to the tip of its dendrite ranged up to 600 μm in the transverse plane (Sholl analysis, Fig. 4C). The thickness of our slices (450–600 μm) would therefore be sufficient to contain the major dendrites in the rostrocaudal direction. Second, if dendritic shafts extending in the rostrocaudal direction (in the Z-axis direction on slices) were cut, their stumps would often have been seen near the somata. This was not the case, however.

In this study MNs were identified based on retrograde labeling from the target muscles. In addition, the cells were individually labeled with injected Neurobiotin. This approach provided two advantages for observing the morphology of MNs. First, Neurobiotin labeled nearly the entire dendritic tree of target MNs. In the past, although retrograde tracers were often used to identify MNs and study their morphology^9^, the tracers used labeled only the somata and the proximal-most dendrites^28^. This may be one reason for the prevailing view that MNs are “stellate”. Second, the morphology of each MN was individually studied. Pectoralis MNs exhibited a morphological spectrum that extended from stellate to bidirectional. However, in a study where a cluster of MNs labeled by a tracer were observed collectively, pectoralis MNs were reported to be “the type that extends dendrites to the gray matter side”^12^. In such cases, dendrites of stellate cells may have overlaid on bidirectional cells masking their morphology.

As mentioned, the three groups of MNs studied each showed characteristic morphologies (Figs. 1, 2, 4). The distally located, forearm-muscle MNs were a hemidirectional multipolar type, while the proximally positioned, scapular-muscle MNs were mostly bidirectional. Interestingly, the dendritic patterns of MNs innervating intermediately-located pectoralis muscles exhibited intermediate morphologies, between the other two groups. MN pools thus showed a spectrum of morphologies that reflect the location of the muscles they innervate along a distal-to-proximal axis. Dendritic orientation determines the selectivity and diversity of the afferents they receive, and the arborization pattern determines whether they receive a dense or sparse sampling of the input information^29^. Scapular MNs exhibited bidirectional dendritic fields along the gray matter boundary, which may be suitable for receiving afferents from the adjacently located vestibulospinal and reticulospinal tracts^30–34^. On the other hand, multipolar fields of forearm MNs may reflect the fact that forearm muscles mainly involved in finer, more dexterous movements require a wider variety of inputs from descending tracts and the periphery. It would be interesting to investigate the organizational differences of inputs to MNs innervating muscles along a distal-to-proximal gradient and its significance to information processing for motor control.

## Methods

### Animals

Experiments were conducted in 49 Wistar rats of both sexes ranging in age from postnatal day 7–9 (P7–P9). All experiments were performed in accordance with National Institutes of Health Guide for the Care and Use of Laboratory Animals (8th edition) and were approved by Institutional Animal Care and Use Committee at Teikyo University School of Medicine (No. 10-007).

### Retrograde labeling of cervical motor neurons

**Figure 5:**
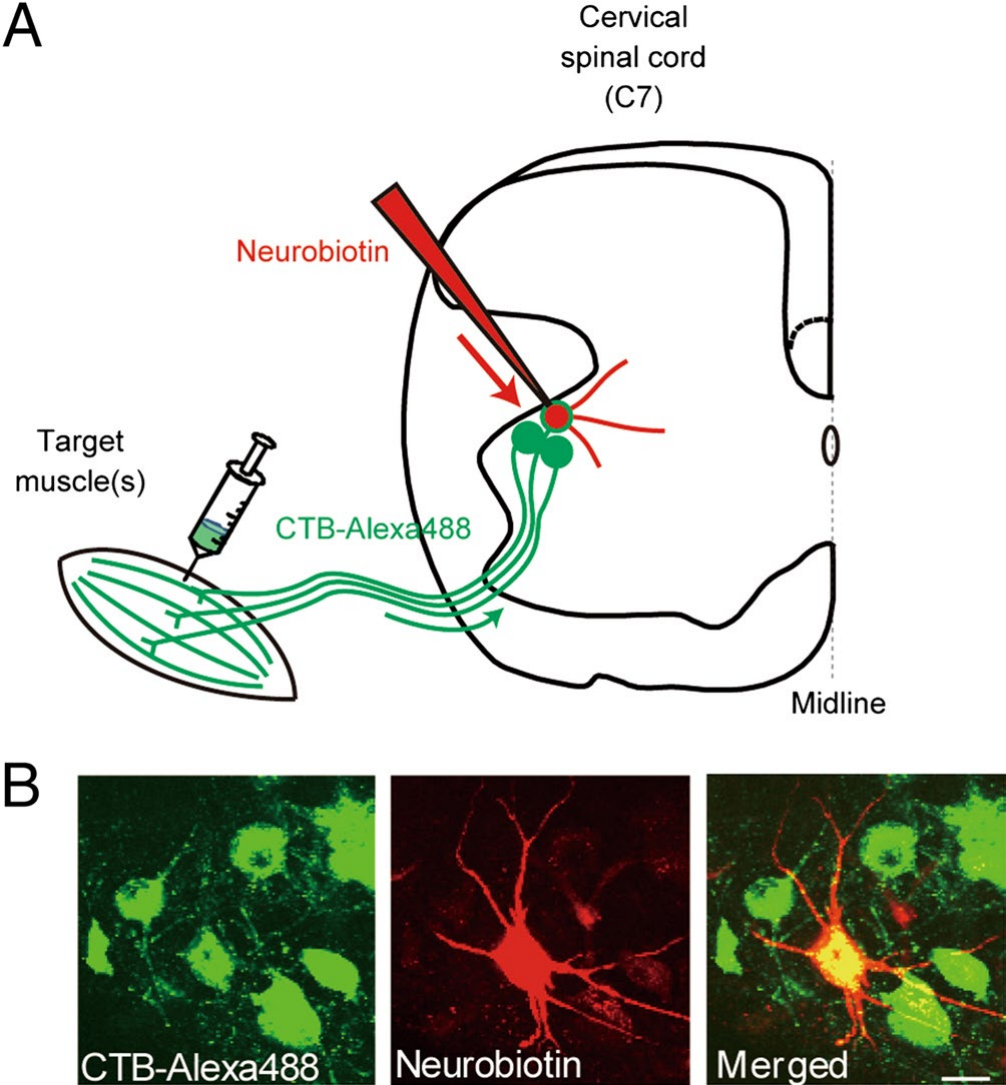
Retrograde labeling of motoneurons (MNs) with CTB and total labeling of dendritic arbors with Neurobiotin. (**A**) Schematic drawing of the experimental arrangement. MNs were retrogradely labeled by CTB-Alexa 488 injected into the target muscles. Transverse cervical spinal cord slices (C6–C8) were prepared from P7–P9 rats. Whole-cell recordings were made from the retrogradely labeled MNs, during which Neurobiotin in the pipette diffused into MNs’ dendrites. (**B**) A MN soma retrogradely labeled green with CTB-Alexa 488 (left), stained red by NeutrAvidin-Texas Red bound to injected Neurobiotin (center), and a merged image (right). Scale bar = 20 μm.

On P4–P6, rats were anesthetized with isoflurane and an incision was made in the skin to expose the target muscles. To retrogradely label defined groups of MNs in the cervical cord, we injected cholera toxin subunit B-conjugated Alexa Fluor 488 dye (CTB-Alexa 488, Life Technologies, Carlsbad, CA, USA). We injected the tracer into the forearm at two or three sites on both the extensor and flexor sides (1 μl × 4–5 sites) in order to label as many muscles as possible, including extensor carpi radialis/ulnaris, extensor digitorum communis, palmaris longus, flexor digitorum profundus, pronator teres, and flexor carpi radialis/ulnaris. However, we were unable to specify all of the muscles injected, as this group contained fine and slender muscles, like extensor digiti quarti and extensor pollicis longus/brevis. We also injected the pectoralis major muscle (1 μl × 4 sites) and the serratus anterior muscle (1 μl × one site) (Fig. 5A). After closing the incisions with cyanoacrylate adhesive, the animals were returned to their dams. MNs were clearly labeled 48–72 h after the procedure^14^. Using a fluorescence microscope, we examined the distribution of injected dye and found that the Alexa 488 fluorescence did not spread outside the targeted muscles (data not shown). However, because the serratus anterior muscle is too small around P8 and the signal from the injected CTB-Alexa 488 was too weak, we could not make reliable observations of the spread of the dye. In a preliminary experiment using an AAV-GFP vector injected into a serratus anterior muscle of the same age, the subscapularis and teres major muscles also showed weak GFP fluorescence on P35 (data not shown). This means dye injected into the serratus anterior muscle may have spread somewhat to the subscapularis and teres major muscles. In this paper we used the term “scapular muscles” for convenience to describe these shoulder muscles, which include the anterior serratus, subscapularis and teres major muscles; but the trapezius and deltoid muscles were not included. The cell bodies of scapular MNs are overwhelmingly located on the ventromedial side of the ventral horn. Although a portion of the MN somata for a scapular muscle (teres major) is reportedly located on the ventrolateral side^15^, we analyzed only the ventromedial group in this study.

### Direct labeling of MNs

Spinal cord slices were prepared from P7–P9 rat cervical cords^13^. Briefly, transverse slices (450–600 μm thick) of C6–C8 cervical segments were cut in oxygenated ice-cold cutting solution using a Linearslicer (PRO7, Dosaka EM, Kyoto, Japan) and transferred to a slice chamber perfused for more than 1 h at 15 ml/min with oxygenated artificial cerebrospinal fluid (ACSF).

Whole-cell currents were recorded from fluorescent MNs using patch-pipettes^13^ (Fig. 5A). The internal solution in the pipettes contained 50 μM N-(2-aminoethyl) biotinamide hydrochloride (Neurobiotin, Vector Laboratories, Burlingame, CA, USA), which was later visualized within MNs by reacting it with Texas Red-avidin (Fig. 5B). The whole-cell configuration was maintained for at least 20–40 min, which allowed the Neurobiotin to diffuse through the entire dendritic tree^16^. Some of the morphological data presented in this study was obtained from common materials in our previous electrophysiological study^13^.

### Staining and morphometric analysis

The spinal cord slices were fixed with 4% paraformaldehyde in 0.1 M sodium phosphate buffer overnight at 4 °C. They were then washed twice for 10 min each in 0.05 M phosphate-buffered saline (PBS) containing 0.1% Triton X-100. Next, the slices were left overnight gently shaking in 0.1% Triton-X PBS containing NeutrAvidin-TexasRed (1:500 dilution, Life Technologies) at 4 °C. After washing three times in PBS, free-floating slices were mounted on slide glasses and analyzed using a two-photon laser scanning microscope (FV1000; Olympus, Tokyo, Japan) (Fig. 5B). Among the 52 cells positive for Neurobiotin fluorescence, 50 were also positive for Alexa 488 fluorescence.

Stacked images of labeled MNs were three-dimensionally traced and reconstructed using Neurolucida software version 11.01.2 (MBF Bioscience, Willston, VT, USA, https://www.mbfbioscience.com/neurolucida). With this software, MNs located on the right side of the spinal cord were geometrically turned over the midline. Morphometric analysis of the reconstructed MNs was performed using Neurolucida Explorer version 11.01.2 (MBF Bioscience) (see “Results” section).

### Statistical methods

All statistical data (Fig. 4) are presented as the mean ± standard error of the mean (SEM). The lengths of dendrites (Fig. 4A, B) and results of Sholl analyses (Fig. 4C) were compared between two groups of pools using Student’s t-test.
